# Comparative genomics of nuclear envelope proteins

**DOI:** 10.1186/s12864-018-5218-4

**Published:** 2018-11-16

**Authors:** Hita Sony Garapati, Krishnaveni Mishra

**Affiliations:** Department of Biochemistry, School of Life Sciences, University of Hyderabad, Hyderabad, 500046 India

**Keywords:** Nuclear envelope, Eukaryotic supergroups, Comparative genomics, LECA

## Abstract

**Background:**

The nuclear envelope (NE) that encapsulates the nuclear genome is a double lipid bilayer with several integral and peripherally associated proteins. It is a characteristic feature of the eukaryotes and acts as a hub for a number of important nuclear events including transcription, repair, and regulated gene expression. The proteins associated with the nuclear envelope mediate the NE functions and maintain its structural integrity, which is crucial for survival. In spite of the importance of this structure, knowledge of the protein composition of the nuclear envelope and their function, are limited to very few organisms belonging to Opisthokonta and Archaeplastida supergroups. The NE composition is largely unknown in organisms outside these two supergroups.

**Results:**

In this study, we have taken a comparative sequence analysis approach to identify the NE proteome that is present across all five eukaryotic supergroups. We identified 22 proteins involved in various nuclear functions to be part of the core NE proteome. The presence of these proteins across eukaryotes, suggests that they are traceable to the Last Eukaryotic Common Ancestor (LECA). Additionally, we also identified the NE proteins that have evolved in a lineage specific manner and those that have been preserved only in a subset of organisms.

**Conclusions:**

Our study identifies the conserved features of the nuclear envelope across eukaryotes and provides insights into the potential composition and the functionalities that were constituents of the LECA NE.

**Electronic supplementary material:**

The online version of this article (10.1186/s12864-018-5218-4) contains supplementary material, which is available to authorized users.

## Background

The presence of the nucleus and other such sub-cellular compartments distinguishes eukaryotes from prokaryotes. These compartments enable eukaryotes to spatially isolate activities including transcription, translation, energy metabolism and other catabolic and anabolic processes. The evolution of an enclosed nucleus separated the various steps in gene regulation and likely contributed to the enormous developmental complexity of eukaryotes. Apart from physically separating the genetic material from the cytoplasm, the nuclear envelope participates actively in multiple nuclear functions. Nuclear envelope plays a key role in the non-random organization of the genome and the interactions of the chromatin with nuclear envelope proteins are crucial for gene regulation, DNA repair and maintaining genome stability. It also serves as an anchor for the centrosome and nucleolus, prominent nuclear structures important for cell division and ribosome assembly respectively [1]. In addition, the nuclear envelope links the chromatin and the nucleus to the cytoskeleton through proteins present in the nucleus, the inner and outer membrane and provides structural integrity to the nucleus [2].

Maintaining the nuclear envelope integrity is essential for genome stability and cell survival. However, during cell division the nuclear envelope undergoes dramatic changes ranging from complete breakdown and reassembly in organisms undergoing open mitosis to morphological changes and expansion in organisms undergoing closed mitosis [3, 4]. The proteins associated with the nuclear envelope regulate remodeling during mitosis. For example, the chromatin interacting proteins such as Lap2β, Man1 and emerin mediate NE reassembly in human cells at the end of mitosis [5, 6] and the localization of SUN domain protein is tightly coupled to NE dynamics during mitosis in *Arabidopsis thaliana* [7]. The biological significance and the functions of the nuclear envelope proteins known so far are from studies limited to fungi, animals and plants that belong to the Opisthokonta and the Archaeplastida supergroups, which are just two of the five eukaryotic supergroups. Knowledge of the nuclear envelope proteins and their functions in organisms belonging to all five supergroups is necessary to understand the fundamental functions of the nuclear envelope common to all eukaryotes and also provide insights into the LECA NE proteome and its functions.

With the availability of genome sequence data, comparative genomic studies across organisms belonging to all five supergroups viz. Opisthokonta, Amoebozoa, Excavata, SAR and Archaeplastida have been performed for a large number of structures and processes. Extensive comparative genomic studies have been carried out for components involved in nucleocytoplasmic transport of proteins; karyopherins [8, 9], RNA export [10], cell division [11] and kinetochores, a key component in chromosome segregation in all eukaryotes [12]. These studies have identified the core machinery that is conserved across all supergroups and likely to have been a component of the LECA. Similarly, comparative studies have been useful in identifying the nuclear structural components across eukaryotes. For example, analysis of the components of nuclear pore complex (NPC) in various organisms including yeast, vertebrates, amoeba and parasites has shown that the NPC and its components are well conserved and most key components existed in LECA [13, 14]. Lamins, key architectural proteins of metazoans, are now considered to be more widely distributed and lamin-like protein in the eukaryotic ancestor has been proposed [15]. Functional lamin homologues have been proposed in plants (NMCP group of proteins) and in Trypanosomes (NUP-1), however, for these proteins the structural and evolutionary relationship with the metazoan lamins is not obvious [16, 17]. In addition, the chromatin interacting nuclear envelope proteins such as LEM and SUN domain proteins have been proposed to be present in LECA [8]. However, many other components of the nuclear envelope have not been analysed for presence in the LECA and therefore, we have no information on the conservation of the overall architecture of the nuclear envelope.

Understanding the evolution of the nuclear envelope proteome will provide insights into the plasticity of this organelle. Comparing nuclear envelope proteome of organisms within supergroups and between supergroups is likely to identify the core group of nuclear envelope proteins that evolved in the early ancestor of eukaryotes. In this study, we attempt to provide a broad picture of the nuclear envelope proteome of the last eukaryotic common ancestor. We used an approach to begin with the nuclear envelope proteome of yeast, the simplest eukaryote with good annotation and then identify potential homologues across eukaryotes using sequence comparison approaches. We thus identified the conserved nuclear envelope proteins across all supergroups of eukaryotes. After identifying potentially conserved nuclear envelope proteins, we took advantage of the annotation data available for animals and plants and asked how many were nuclear envelope proteins. Our result shows that a large number of them are found in the nuclear envelope of these organisms and also perform similar functions. Therefore, these proteins were likely constituents of the NE of the eukaryotic ancestor. Through this analysis we contribute to our understanding of the critical components that provide the complexity to the nuclear membrane.

## Results

Our goal in this analysis was to identify the evolutionarily conserved proteins of the nuclear envelope. To do this we first selected the nuclear envelope proteins of *Saccharomyces cerevisiae* based on the available sub-cellular localization data. Forty-five proteins localizing to the INM/ONM of *Saccharomyces cerevisiae* were selected as queries for analysis (Additional file 1: Table S1). The nuclear pore complex proteins were excluded from analysis as they were earlier shown to be present in LECA [13, 14]. The selected proteins fell into four broad functional classes, namely, chromatin organization, nuclear envelope homeostasis, gene regulation and transport. Proteins whose function either did not fit into any of the four categories or whose function is unknown were grouped into “others category”. The homologs of the nuclear envelope proteins were identified from 73 eukaryotes belonging to the 5 eukaryotic supergroups (Additional file 1: Table S2). The classification of the eukaryotic supergroups and the relationship between the organisms is adopted from phylogenomic studies [18–23]. The proteins included in the study have varying degrees of conservation, with some proteins being highly conserved to some that are rapidly evolving. In order to maximize the detection of homologs across distantly related organisms, we built profile HMMs from homologs detected in closely related organisms and used them to identify the homologs in the 73 proteome datasets (see methods). We mapped the presence/absence of the homologs across the 73 eukaryotic lineages (Additional file 2).

In this study, we identified 22 nuclear envelope proteins that are found in at least one organism across the five eukaryotic supergroups termed the “core proteins”. For a subset of proteins (10 out of 45), homologs were identified in more than one but not in all supergroups. Such proteins are termed as “non-linearly conserved proteins”. In addition, as we started our analysis with the budding yeast nuclear envelope proteome, we identified proteins whose homologs are restricted to fungi termed the “fungal specific proteins”. The failure to detect a homolog does not necessarily indicate absence in those organisms, as it is possible that the protein has diverged extensively in those lineages and could not be detected in our homology searches. Our analysis, therefore, identifies the minimal core and the lineage specific NE components and provides insights into the probable composition of the LECA nuclear envelope.

### Chromatin organization

**Fig. 1:**
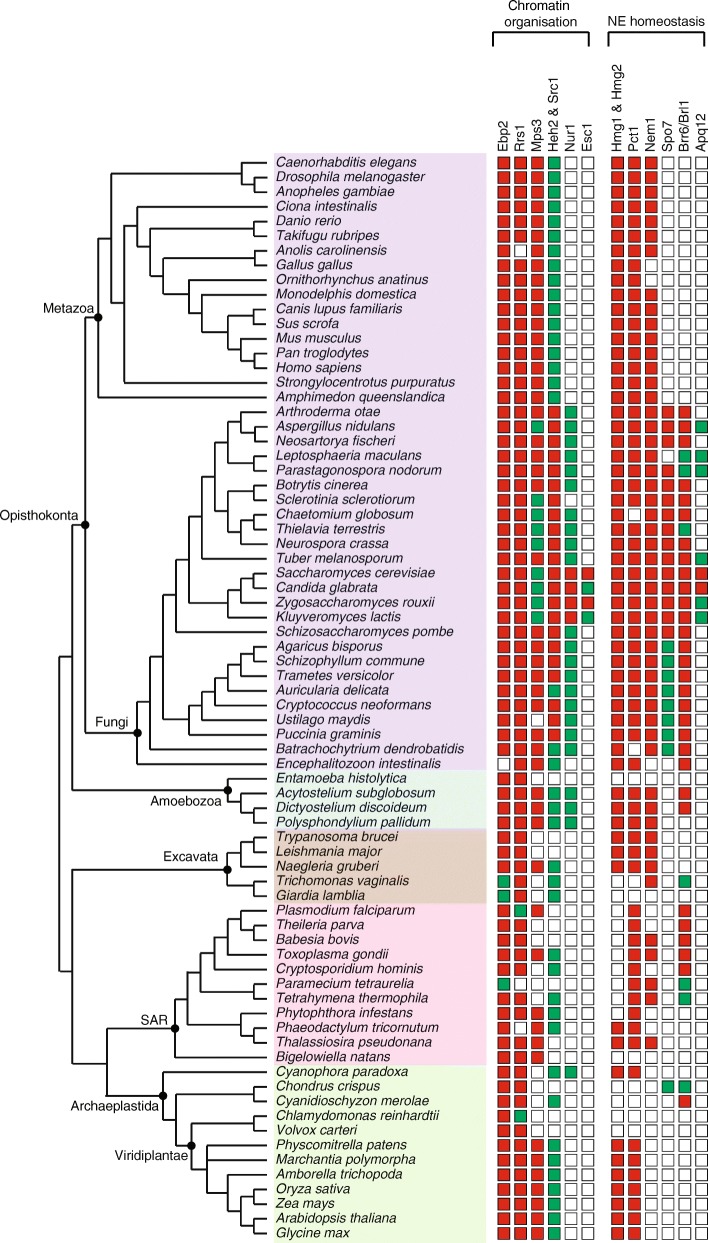
Proteins involved in chromatin organization and NE homeostasis across eukaryotes. The presence/absence and the degree of conservation of homologs identified for chromatin organization and NE homeostasis proteins are shown. Red filled squares represent the homologs validated using rBLAST with significant E-value (less than 10^− 5^). The green-filled squares represent the homologs that can be found only using *hmmsearch* and share conserved region/domain. The supergroups Opisthokonta, Ameobozoa, Excavata, SAR and Archaeplastida are shaded in purple, blue, brown, pink and green filled rectangles, respectively

Nuclear envelope proteins, specifically the ones at the INM play a crucial role in the dynamic organization of the chromatin into active and repressive domains in many eukaryotes [1, 24]. The budding yeast nuclear envelope consists of about 7 proteins that are involved in clustering of telomeres and/or anchoring of the telomeres and rDNA to the nuclear periphery. These include Ebp2, Rrs1, Mps3, Heh2, Src1, Nur1 and Esc1. We find that 5 of these proteins, namely, Mps3, Heh2, Src1, Ebp2 and Rrs1, were found across all supergroups and are part of the core NE proteome (Fig. 1), although the degree of conservation is variable. Ebp2 and Rrs1 proteins are highly conserved proteins and their homologs were found in most of the organisms considered in this study, while the homologs of the SUN domain protein Mps3, although well-conserved, could not be detected in a few bikonts. Mps3 homologs in Saccharomycetes have diverged significantly from the rest of the eukaryotes (Fig. 1). In rBLAST analysis, most of the homologs identified return the *S. pombe* SUN domain protein with significant E-value but not the Mps3 of *S. cerevisiae*.

For the two paralogous proteins Heh2 & Src1, even though the conserved domains were found across supergroups, the overall conservation was relatively low. The homologs of Heh2 and Src1 proteins identified share homology at the MSC domain located at the C-terminal. A lineage specific additional domain could be detected in opisthokonts: the homologs in fungi have HeH domain at the N-terminal, while the animal homologs have a LEM domain. Outside opisthokonts, an N-terminal HeH domain in combination with MSC domain is found only in *N. gruberi*, an Excavate (Additional file 1: Figure S1).

The homologs of two of the chromatin interacting proteins Nur1 and Esc1 are lineage specific. Nur1 was found to be a non-linearly conserved protein; the homologs of Nur1 could be detected only in fungi, mycetozoa and the glaucophyte, *C. paradoxa.* The homologs outside Saccharomycetes do not share significant sequence similarity with the Nur1 protein. The homologs of Esc1 protein were restricted to Saccharomycetes and share homology in only a small region.

### Nuclear envelope homeostasis

The shape of the nuclear envelope and its dynamics are coupled to the genes regulating lipid synthesis and maintaining lipid homeostasis. Several of the proteins encoded by these genes are found as integral membrane proteins of the NE-ER in yeast and include the paralogous sterol synthesis genes Hmg1, Hmg2 [25]; regulators of phospholipid biosynthesis Nem1, Spo7, Pct1 [26] and the genes that maintain lipid homeostasis Brr6, Brl1 and Apq12 [27]. Of these, Hmg1 & Hmg2, Pct1, Brr6 and Brl1 are part of the core NE proteome. While, homologs of HMG-CoA reductase are extensively present across opisthokonts, they could not be found in a number of organisms in SAR (Fig. 1). The homologs of these proteins in opisthokonts have HMG-CoA_red and Sterol_sensing domains, while in other supergroups only the HMG-CoA_red domain is present, suggesting the addition of the Sterol_sensing domain in the ancestor of opisthokonts. Further, the fungal homologs of Hmg1 and Hmg2 have an additional HPIH domain at the N-terminus (Additional file 1: Figure S2). Among the phospholipid biosynthesis genes, Pct1 is found in all supergroups, Nem1 is present in all four supergroups except for Archaeplastida. In ciliates, we find an expansion of the Nem1 homologs, with *T. thermophila* having 3 and *P. tetraurelia* having 22 homologs. In our study, Spo7 homologs are found only in fungi and in one red alga. However, previous studies have shown the presence of a Spo7 ortholog in mammals, which could be identified using the *S. pombe* Spo7, but not *S. cerevisiae* Spo7 [28]. Interestingly, we find that the homologs of Brr6/Brl1 are restricted to only a few organisms across all five supergroups. They were found only in fungi in Opisthokonta, slime molds in Amoebozoa, parabasalids in Excavata, alveolates in SAR and rhodophytes in Archaeaplastida. This suggests secondary loss in large subsets of organisms across supergroups. The homologs of Apq12 protein are present only in ascomycetes and share very low sequence similarity.

### Gene regulation

**Fig. 2:**
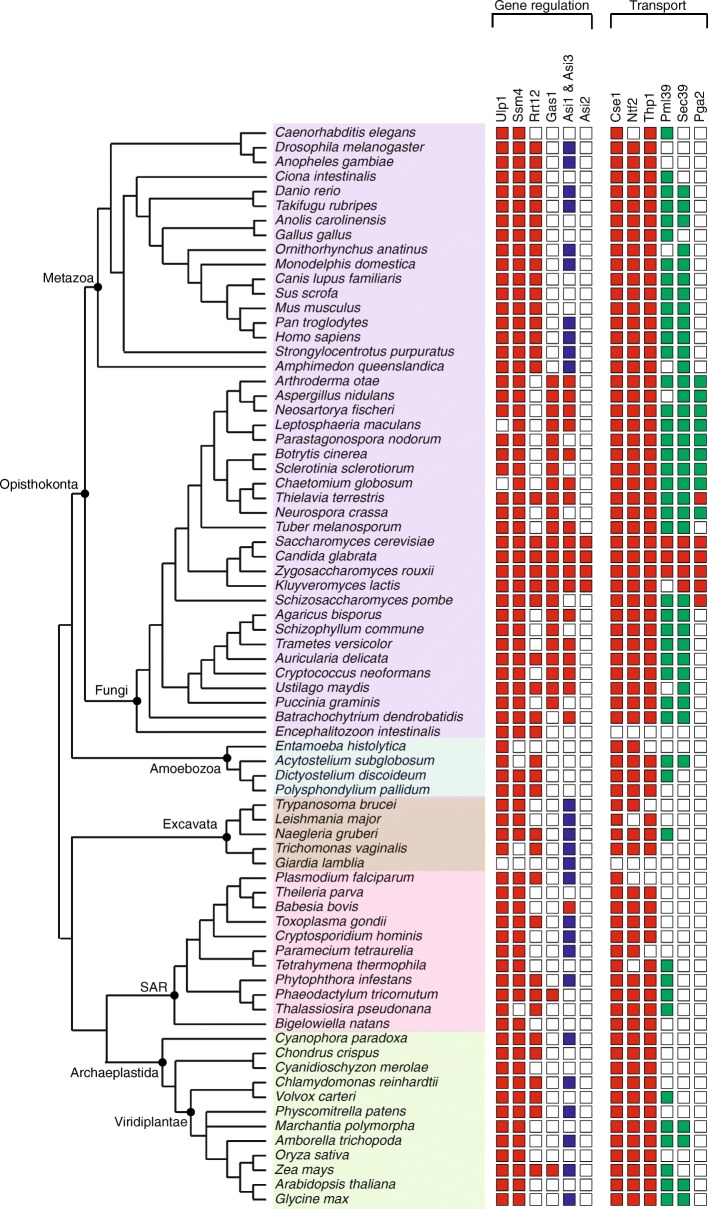
Gene regulation and transport proteins across eukaryotes. The presence/absence and the degree of conservation of homologs identified for proteins involved in gene regulation and transport are shown. Red filled rectangles represent the homologs validated using rBLAST with significant E-value (less than 10^–^^5^). Blue filled rectangles represent the homologs validated using rBLAST but with E-value higher than 10^–^^5^ but less than 10^− 2^. The green filled rectangles represent the homologs that can be found only using *hmmsearch* and share conserved region/domain. The supergroups Opisthokonta, Ameobozoa, Excavata, SAR and Archaeplastida are shaded in purple, blue, brown, pink and green filled rectangles, respectively

Some proteins present at the inner nuclear membrane contribute to the spatio-temporal regulation of gene expression. This regulation is achieved by the post-translational modification of the transcription activators/repressors that are targeted to the nuclear envelope. The INM of yeast hosts proteins like Ulp1 (SUMO protease), Ssm4, Asi1 & Asi3 (Ubiquitin ligases), Rrt12 (peptidase) and Gas1 (1,3-beta-glucanosyltransferase) that regulate gene expression [29–33]. Homologs of Ulp1, Ssm4 and Rrt12 are found across all eukaryotic supergroups (Fig. 2). The human Ulp1 is also associated with the NPC and the homolog of Ssm4 in human is found in ER; while in yeast it is both at INM and ER (Additional file 3). Asi1 and Asi3 are categorized as non-linearly conserved proteins as no homolog could be detected in amoeba. The fungal homologs and the Apicomplexan *B. bovis,* share significant sequence similarity with Asi1/Asi3; however, most of the others return Asi1/Asi3 as top-most hits in rBLAST but with an E-value higher than 10^− 5^ but less than 10^− 2^. Asi2 protein, which works together with Asi1 & Asi3 proteins, is present only in Saccharomycetes. The homologs of the Gas1 protein were found in fungi, Bacillariophyta and in *Zea mays*. Remarkably, the homolog in *Zea mays* shares the same domain architecture and significant sequence similarity with yeast Gas1.

### Transport

The nuclear pore proteins embedded into the nuclear envelope mediate the nucleocytoplasmic transport of macromolecules and are conserved across eukaryotes. Though pore complex proteins were excluded from our analysis, we did consider a few pore-associated proteins namely, Cse1 and Ntf2, that are involved in nucleocytoplasmic transport of the proteins [34, 35]; Thp1, involved in mRNA export [36] and Pml39, involved in retaining unspliced mRNAs inside the nucleus [37]. Additionally, two proteins, Sec39 and Pga2, proteins involved in vesicle-mediated transport and protein processing/trafficking, respectively, localized to nuclear membrane/ER [38, 39]. The proteins that are part of the core proteome in this category include Cse1, Ntf2, Thp1 and Pml39. While, the homologs of Cse1, Ntf2 and Thp1 are well conserved across eukaryotes, the Pml39 homologs outside Saccharomycetes do not show good conservation and cannot be identified by BLASTp (Fig. 2). Thp1 homologs identified across eukaryotes showed good conservation with the *A. delicata* Thp1 homolog rather than the *S. cerevisiae* Thp1 in rBLAST. This suggests divergence in Saccharomycetes homologs. Sec39 is categorized as a non-linearly conserved protein as homologs were found only in opisthokonts, amoeba and plants. Pga2 is a fungal-specific protein whose homologs were found only in ascomycetes.

### Others

**Fig. 3:**
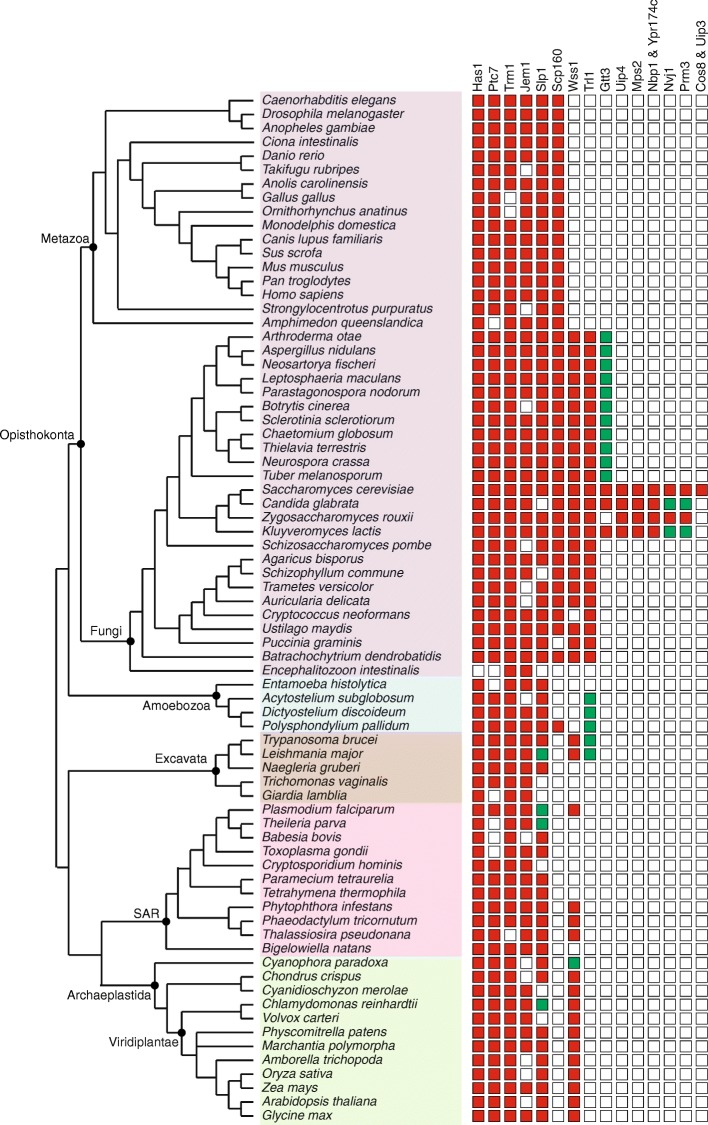
Other NE proteins found across eukaryotes. The presence/absence and the degree of conservation of homologs identified for proteins categorized under “others” are shown. Red filled rectangles represent the homologs validated using rBLAST with significant E-value (less than 10^− 5^). The green filled rectangles represent the homologs that can be found only using *hmmsearch* and share conserved region/domain. The supergroups Opisthokonta, Ameobozoa, Excavata, SAR and Archaeplastida are shaded in purple, blue, brown, pink and green filled rectangles, respectively

Apart from the proteins belonging to the above mentioned functional categories, we find proteins with diverse functions and some with yet unknown functions associated with the nuclear envelope. While, some of these proteins are part of the core protein group, a large number of these are found to be fungal specific (Fig. 3). The core proteins include the helicase, Has1; phosphatase Ptc7; tRNA methyltransferase Trm1; DnaJ chaperone Jem1 and the mid-SUN domain protein Slp1. Has1 and Trm1 are highly conserved overall at the sequence level, while Jem1 homology is limited to the DnaJ domain. The RNA binding protein Scp160, metalloprotease Wss1 and the tRNA ligase Trl1, are classified as non-linearly conserved proteins. Interestingly, a large number of proteins in this category namely, Gtt3, Uip4, Mps2, Nbp1, Ypr174c, Nvj1, Prm3, Cos8 and Uip3 are found only in ascomycetes.

#### Core nuclear envelope proteins

Of the 45 NE proteins analyzed, 22 of them are found in at least one organism in each of the eukaryotic supergroups. These 22 proteins constitute the core nuclear envelope proteome that was probably part of the LECA. Of note, a significant number of proteins that are involved in chromatin organization, NE homeostasis, gene regulation and transport are part of the core proteome. The proteins that are involved in chromatin organization and nuclear envelope homeostasis are also important for maintaining the nuclear architecture in yeast [27, 40–43]. Interestingly, we find that two SUN domain proteins viz., the C-terminal (Mps3) and mid-SUN (Slp1) domain proteins are part of the core proteome (Additional file 1: Figure S3). The mid-SUN domain proteins have expanded in plants and contribute to maintenance of nuclear morphology in *A. thaliana* [44]. Thus, the origin and evolution of the two SUN domain families, appears to predate LECA. This suggests that LECA possessed a sophisticated nuclear envelope proteome that mediated various critical nuclear functions. The conservation of NE proteins that anchor chromatin and enzymes that modulate transcription factors suggest that function of NE as a key architectural component in gene regulation is ancient and potentially existed in LECA. Similarly the presence of the ubiquitin and sumoylation components at the NE suggests an evolutionarily conserved mechanism to maintain nuclear protein homeostasis.

**Fig. 4:**
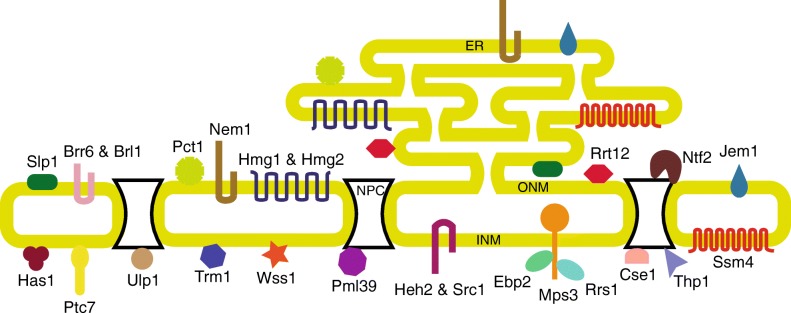
LECA nuclear envelope proteome. A pictorial representation of the LECA nuclear envelope proteome. The core nuclear envelope proteins identified and the non-linearly conserved proteins present in at least 4 eukaryotic supergroups are represented in different shapes and colors based on their available localization data in *S. cerevisiae*. The proteins present at ER-ONM network are shown both at the ER and ONM

The homologs of 10 proteins could not be obtained across all eukaryotic supergroups (Additional file 1: Figure S4). These are termed the non-linearly conserved proteins. Two of these proteins, Nem1 and Wss1 are found in four of the supergroups and could not be detected only in Archaeplastida and Ameobozoa supergroups, respectively. We speculate that these two proteins were probably present in LECA and were lost in some lineages later. Among the other non-linearly conserved proteins, Gas1 homologs are predominantly found in fungi and are found only in *P. tricornutum* and *Z. mays* outside fungi. The presence of these homologs outside fungi is possibly due to an HGT event. Similarly, Nur1, which is present in fungi and Amoeba, which are unikonts is found only in one organism in bikonts viz. *C. paradoxa*. Thus, 24 proteins that are present in all or at least four supergroups probably were constituents of the LECA nuclear envelope proteome (Fig. 4).

#### Fungal specific NE proteins

Out of the 45 proteins used for query, 13 are found only in the fungal kingdom, the ascomycetes, and among them 10 are found only in Saccharomycetes. Among the Saccharomycetes specific proteins, a majority are rapidly evolving and the homologs share very low sequence similarity amongst them. We further analysed these sequences to see if there were any conserved motifs and identified short conserved motifs in three of them. One motif each was identified in the N-terminal region of Esc1 protein, C-terminal of Nvj1 and Prm3 proteins (Additional file 1: Figure S5). These motifs were used to mine homologs in other fungi; however, no additional homolog could be identified outside Saccharomycetes.

The identified motifs coincide with regions experimentally tested for function in two proteins. Nvj1 forms nucleus-vacuole junctions through its interaction with Vac8 and promotes piecemeal microautophagy of the nucleus [45]. The motif identified overlaps with the region that was earlier shown to be sufficient and necessary for interaction with Vac8 [46] suggesting that this function is likely conserved across Saccharomycetes. Prm3 protein plays an important role in nuclear fusion event, which is the final step in yeast mating pathway. The motif identified is part of the region that was shown to be important for stability, localization and function of this protein [47]. While most of these genes are non-essential for the survival of yeast, only Nbp1, which is required for the insertion of spindle pole body into the nuclear membrane, is essential. The presence of around 20% of NE proteome unique to Saccharomycetes is an indication of the fast evolving and plastic nature of the nuclear envelope proteome.

## Discussion

Our comparative genomic study of nuclear envelope proteins in eukaryotic supergroups has identified a set of 24 proteins of which 22 are present in all supergroups and 2 in four of the supergroups. Of the 24, 10 localize to NE/ER in either human/mouse/Arabidopsis (Additional file 3). We speculate that these were likely components of the early ancestor of eukaryotes, the LECA and perhaps carry out similar functions in all organisms including LECA. This comprehensive analysis serves as a starting point to understand the composition and complexity of the ancestral nuclear envelope. The NE in extant eukaryotes is a physical barrier that separates the genome from the rest of the components. The NPC are thought to have coevolved with the NE to allow transport of molecules between the cytoplasm and nucleus. The NE also partakes in essential functions like maintenance of nuclear architecture, chromatin organization, control of transcription and DNA repair. Proteins and protein complexes that mediate these functions are found either as associated with or integrated in the nuclear envelope in yeast and animals. In our analysis we find that proteins involved in these processes are conserved across supergroups.

In *S. cerevisiae,* loss of the chromatin interacting proteins of the core proteome, leads to nuclear morphology defects. The function of some of these proteins is conserved across eukaryotic supergroups. For example, the C-terminal SUN domain protein in *S. cerevisiae* is required for SPB duplication and insertion into the nuclear envelope [48], while the ortholog identified in the evolutionarily distant amoeba, *D. discoideum* maintains the connection between centrosome and nuclear envelope through its interaction with chromatin [49]. In addition, the C-terminal SUN domain proteins in yeast, animals and plants are known to tether telomeres to the nuclear periphery during meiosis [41, 50]. The paralogous proteins Heh2 and Src1 in yeast tether telomeres and rDNA to the nuclear periphery. The orthologs of these proteins in *S. pombe* and in human are critical for maintaining nuclear envelope morphology through their interactions with chromatin [42, 51]. Positioning of chromosomes in the nucleus, which in turn influences gene expression, is regulated through interaction with these nuclear envelope proteins. This suggests an early evolution of the chromatin-NE interaction and consequent gene regulation mechanisms.

Two proteins, Ebp2 and Rrs1 involved in ribosome biogenesis and telomere clustering in *Saccharomyces cerevisiae* [40] are present in almost all the organisms considered in this study. The human Rrs1 ortholog also contributes to proper separation of the chromosomes during mitosis in addition to regulating the ribosome synthesis [52]. This suggests a conserved function of the Rrs1 protein in chromatin interaction in opisthokonts. As more functional data from eukaryotes become available we would know if the chromatin interaction in ribosome biogenesis proteins is ancient or a feature evolved only in opisthokonts.

Another important class of proteins conserved across supergroups are the SUMO proteases and ubiquitin ligases. The SUMO protease Ulp1, is associated with nuclear pores in *S. cerevisiae* where it desumoylates, among others, specific transcription activators and repressors and regulates the transcription of genes in an NPC dependent manner [29]. One of the Ulp1 orthologs in *Arabidopsis thaliana* Esd4, also identified as a part of the core proteome in this study, localizes to the nuclear periphery and the mutants have low levels of a transcription factor which acts as repressor for flowering [53, 54]. Similarly, the Ubiquitin ligase Ssm4, present at the INM in yeast, degrades the transcription factor matα2 that represses a-specific genes in α cells [30, 55, 56]. We find orthologs of Ssm4 across all eukaryotes and the ortholog in *Arabidopsis*, Sud1 is found to regulate HMG-CoA reductase activity [57]. However, the mechanism of this regulation is still unknown. Together, these data indicate that the SUMO and ubiquitin mediated protein homeostasis is a conserved function associated with the nuclear envelope. Since this is found in both unikonts and bikonts, we speculate that this property evolved in the ancient nuclear envelope.

A significant number of proteins that are involved in lipid biosynthesis are found to be part of the LECA nuclear envelope proteome. The nuclear envelope expansion during cell division [3, 4] requires additional nuclear membrane synthesis that is regulated by the proteins associated with the ER-ONM network. The Nem1-Spo7 phosphatase complex in yeast dephosphorylates the PA phosphatase, Lipin/Pah1, that mediates the conversion of phosphatidic acid (PA) to diacylglycerol (DAG) and thus restrict membrane growth. On the other hand, the phosphorylation of Pah1 allows the nuclear membrane growth [26]. The human Nem1 ortholog, Dullard, is an NE protein and ectopic expression in yeast rescues the NE defects of *nem1Δ* cells [58, 59]. Recent studies demonstrated Pah1 and Nem1 mediated regulation of lipid droplet number in the ciliate *Tetrahymena thermophila* [60]. This suggests the presence of a conserved mechanism for regulating lipid biosynthesis and membrane homeostasis across eukaryotes. Pct1 gene involved in phosphotidylcholine synthesis and HMG-CoA reductase involved in sterol biosynthesis are found in organisms across all supergroups. In yeast, over-production of Hmg1 leads to karmellae formation [43]. Similarly, the deletion of HMG-CoA reductase in Arabidopsis leads to altered ER morphology around the nucleus [61]. In *S. cerevisiae*, the proteins Brr6, Brl1 and Apq12 are integral membrane proteins and form a complex. The mutants of these proteins are found to have altered lipid composition in membranes along with defects in nuclear envelope morphology and NPC biogenesis [27, 62]. Although we could not detect Archaeplastida homologues for Nem1, or Pct1 and Hmg1/2 in algae and the Brr6/Brl1 are less widely distributed among members of the supergroups, association of proteins regulating membrane biosynthesis is a widely conserved feature of nuclear envelopes of most eukaryotes.

Another important finding from this study is the identification of 13 proteins specific to ascomycetes, potentially appearing after the Ascomycota-Basidiomycota split. Of the 13 specific to ascomycetes, 10 are restricted to Saccharomycetes. As most of these proteins are found to be rapidly evolving, it is possible that the homolog in organisms outside ascomycetes have diverged to an extent that they cannot be identified by sequence based searches. Nevertheless, the presence of around 20% of NE proteome unique to Saccharomycetes is an indication of the fast evolving and plastic nature of the nuclear envelope proteome. An early proteomic study revealed that there are over 60 nuclear envelope proteins in animals [63, 64] suggesting that the nuclear envelope proteome has undergone tremendous expansion. These data hint at the potential for multiple NE proteins specific to each lineage to have evolved.

This study presents a comprehensive picture of the ancient nuclear envelope proteome. However, there are some limitations. One, many eukaryotes and especially yeasts, have undergone reductive evolution, and therefore, many NE proteins, originally part of LECA NE, may have been lost in yeast but present in other organisms. These would not be identified in this study. Second, there are limitations of sequence-based methods for capturing homologs. Though careful analysis with stringent cut-offs was performed, the identified homologs may still contain some false positives (a protein which is not a homolog) and/or false negatives (failure to detect a homolog). As many proteins included in the analysis are rapidly evolving, there is a high chance for false negatives being present. For example, while no homolog for Spo7 could be identified in Metazoa in this study using the *S. cerevisiae* protein sequence, homology searches using the *S. pombe* protein sequence did find a Spo7 ortholog [28]. Similarly, a more significantly diverged counterpart of Wss1, Spartan, was identified in mammals recently [65]. The failure to detect homologs because of sequence divergence in such cases would falsely implicate gene loss and may also lead to under-representation of genuinely conserved proteins. Using multiple experimental datasets for NE to start this search would be more comprehensive; however, this sort of data is not available currently. Despite these caveats, this study serves as a first step towards reconstructing the LECA NE proteome. With further experimental evidence of NE proteins from diverse organisms we would be able to build a complete picture of this key evolutionary innovation.

## Conclusions

NPCs and a subset of NE proteins have been shown to be present in LECA. However, to date a comprehensive analysis of the NE proteome across a wide range of organisms has not been done. Using comparative genomics approach we identified the core nuclear envelope proteins that are present across all eukaryotic supergroups, the non-linearly conserved and the fungal specific NE proteins. A significant number of proteins involved in chromatin organization, nuclear envelope homeostasis, gene regulation and transport are found to be part of the core proteome, suggesting that they are conserved NE functions that were present in LECA. This study throws light on the fundamental functions of the nuclear envelope and also underscores its plastic nature. As more experimental data from diverse organisms becomes available, this study along with other similar studies will help in understanding the origin and evolution of the nucleus.

## Methods

### Data set preparation

The proteins at the nuclear envelope of *Saccharomyces cerevisiae* were retrieved using a perl script based on the presence of keywords “nuclear envelope”, “nuclear periphery”, “nuclear membrane” in the description of genes in SGD and the localization data in Yeast GFP fusion localization database [66]. The retrieved proteins were further analyzed manually and the nuclear pore complex proteins and spindle pole components were excluded. Finally, 45 NE proteins were considered for the analysis (Additional file 1: Table S1). Throughout the manuscript, unless stated otherwise, the proteins are referred to by their *S. cerevisiae* names.

To identify the homologs of the NE proteins across eukaryotes, 73 eukaryotic species belonging to diverse phyla within the five supergroups (Opisthokonta, Amoebozoa, Excavata, SAR and Archaeplastida) with complete genome sequences were chosen (Additional file 1: Table S2). Preference was given to organisms that are included in RefSeq database and that are used as models. Relatively, a large number of organisms were chosen from fungi (at least two from each class) to study the in-depth distribution patterns of fungal specific NE proteins. The proteomes of all the organisms considered in this study were downloaded from NCBI except for *Bigelowiella natans* and *Cyanophora paradoxa* which were downloaded from JGI genome portal [67] and the Cyanophora Genome Project hosted on the Rutgers University website respectively [68].

### Homolog identification

The homologs of the 45 nuclear envelope proteins across the 73 eukaryotic species were identified using HMMER. Unless specified, all analyses were performed using default parameters of the respective software versions mentioned. In order to build the profile HMMs, for each of the NE proteins, homologs in opisthokonts with E-value less than 10^− 10^ were first retrieved using online PSI-BLAST (3 rounds of iteration against nr database) with the yeast protein as query [69]. The paralogous proteins that arose by gene duplication in *S. cerevisiae* were analyzed together. The retrieved homologs were subjected to multiple sequence alignment using ClustalX version 2.1 [70]. The non-conserved regions of the multiple alignment were trimmed off manually using Jalview (version 2.9) [71]. The conserved region(s) obtained from multiple alignment was then converted into a profile HMM using *hmmbuild* (www.hmmer.org, version HMMER 3.0) [72]. The profile HMM generated was used to search the proteomes of each of the 74 organisms (including *S. cerevisiae*) using *hmmsearch* (version HMMER 3.1b2) with an E-value cut-off of 0.01. The homologs identified using *hmmsearch* were further assessed using reciprocal BLAST searches against the *S. cerevisiae* genome (online BLASTp version 2.7.1 against nr database restricted to *Saccharomyces cerevisiae* S288c sequences) and by looking for the presence of conserved domains using *hmmscan* (version HMMER 3.1b2) with GA cutoffs option against Pfam database (version 28.0). The homologs for which no domains could be detected were further scanned using CD-search at NCBI [73].

When multiple homologs sharing the same conserved region/domain were obtained in the *hmmsearch*, the homolog(s) that returned the *S. cerevisiae* query protein as the top-most hit with an E-value less than 10^− 5^ in rBLAST were considered. A few proteins do not return the *S. cerevisiae* protein with significant E-value in rBLAST; possibly due to extensive sequence divergence, however they do contain the conserved region. For such proteins, as only a single hit was obtained, the homolog from *hmmsearch* was directly considered.

### Motif analysis

For proteins whose homologs were found only in Saccharomycetes, motif analysis was carried out using MEME (version 4.11.2) by setting the minimum and maximum motif width to 6 and 50 respectively and by allowing one occurrence per sequence [74]. The motifs identified were converted into profile HMMs using *hmmbuild* and searched in the proteomes of the fungi using *hmmsearch*.

### Localisation of NE protein homologs

The localization data for the homologs of the LECA NE proteins in, *Mus musculus*, *Homo sapiens* and *Arabidopsis thaliana* were obtained from NCBI. Only ones with experimental evidence of nuclear envelope/nuclear pore/ER membrane localization have been considered.

## Additional files

Additional file 1: **Figure S1.** Domain organization in Heh2 and Src1 proteins. **Figure S2. ** Domain organization in Hmg1 & Hmg2 proteins. **Figure S3.** Domain organization in the SUN domain proteins. **Figure S4.** Non-linearly conserved proteins. **Figure S5.** Motifs identified in Saccharomycetes specific proteins. **Table S1.** Nuclear envelope proteome of *Saccharomyces cerevisiae*. **Table S2.** List of organisms used in the study. (PDF 758 kb)

Additional file 2: Homologs of yeast NE proteins across eukaryotes. The homologs of each of the 45 NE proteins across the 74 organisms, along with the GeneID, protein accession, and the various domains (with coordinates) found are shown. Details of each protein are shown in separate sheets of the excel file. The organisms that are not included in RefSeq are represented with their corresponding fasta headers. The homologs in which the conserved regions could not be detected using Pfam, but found using CD-search are mentioned as CDsearch in the brackets following the coordinates. The additional domains found in some of the homologs are bunched under “Others” and their coordinates are mentioned in the respective columns. (XLSX 185 kb)

Additional file 3: Localization status of conserved NE homologs. The sub-cellular localization data of the homologs of conserved NE proteins is shown in human (*H. sapiens*), mouse (*M. musculus*) and plant (*A. thaliana*) in separate sheets of the excel file. First column gives the conserved NE protein (yeast gene name), the second column lists the gene ID of the homolog of the respective protein found in the respective organism. The following columns give the taxonomy ID of the organism, the gene ID of the homolog (repeated), the gene ontology ID, kind of evidence, gene ontology term and the pubmed ID of the evidence. The rows with text highlighted in red and filled in yellow are the ones with experimental evidence, while those not filled with yellow are annotated as NE/ER although no direct experimental evidence is available. (XLSX 47 kb)

## Abbreviations

DAG: Diacylglycerol
ER: Endoplasmic reticulum
INM: Inner nuclear membrane
LECA: Last eukaryotic common ancestor
NE: Nuclear envelope
NPC: Nuclear pore complex
ONM: Outer nuclear membrane
PA: Phosphatidic acid
