# Supplementary material for: Comparative genomics of nuclear envelope proteins
Source: BMC Genomics. 2018 Nov 16;19:823. doi: 10.1186/s12864-018-5218-4 (PMC6240307; doi:10.1186/s12864-018-5218-4)
Supplement: Supplementary file 1 — Figure S1. Domain organization in Heh2 and Src1 proteins. Figure S2. Domain organization in Hmg1 & Hmg2 proteins. Figure S3. Domain organization in the SUN domain proteins. Figure S4. Non-linearly conserved proteins. Figure S5. Motifs identified in Saccharomycetes specific proteins. Table S1. Nuclear envelope proteome of Saccharomyces cerevisiae. Table S2. List of organisms used in the study. (PDF 758 kb) [file 12864_2018_5218_MOESM1_ESM.pdf]

Additional file 1:

Figures S1 - S5

Tables S1, S2

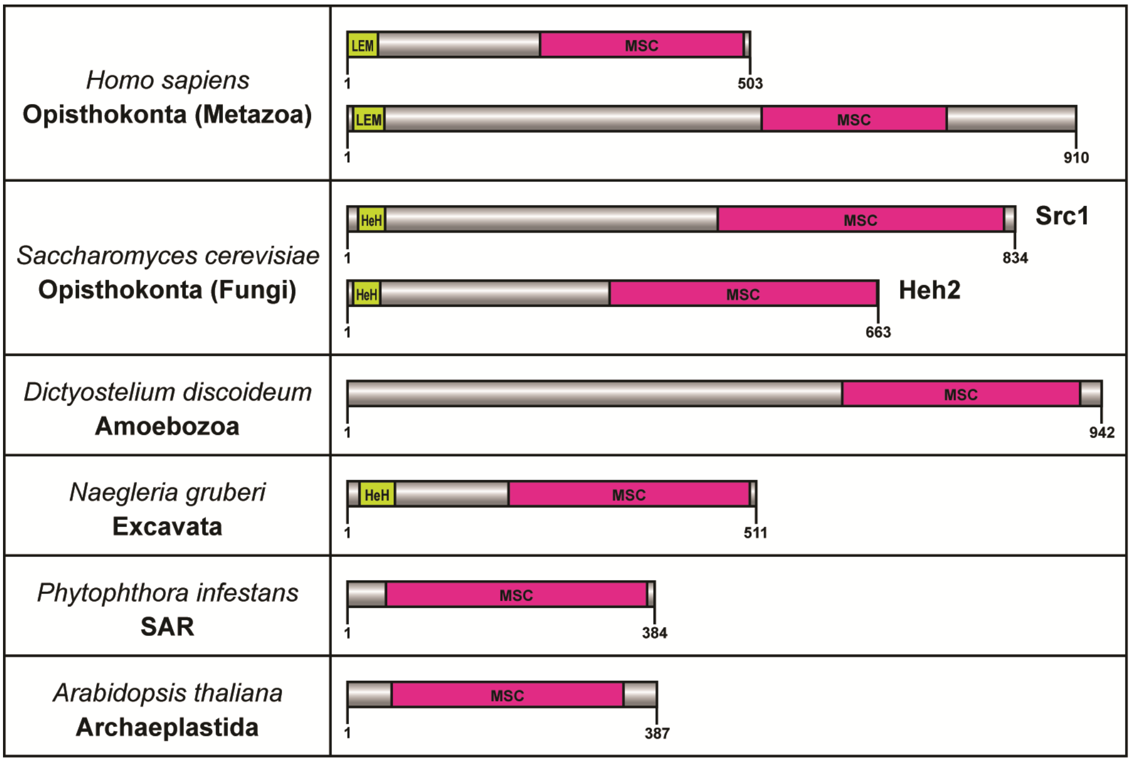

Figure S1: Domain organization in Heh2 and Src1 proteins. The domain architectures of the homologs of Heh2 and Src1 proteins are shown in representative organisms from each eukaryotic supergroup. MSC domain is shown in pink and the N-terminal HeH/LEM domain is shown in green. All maps are drawn to scale.

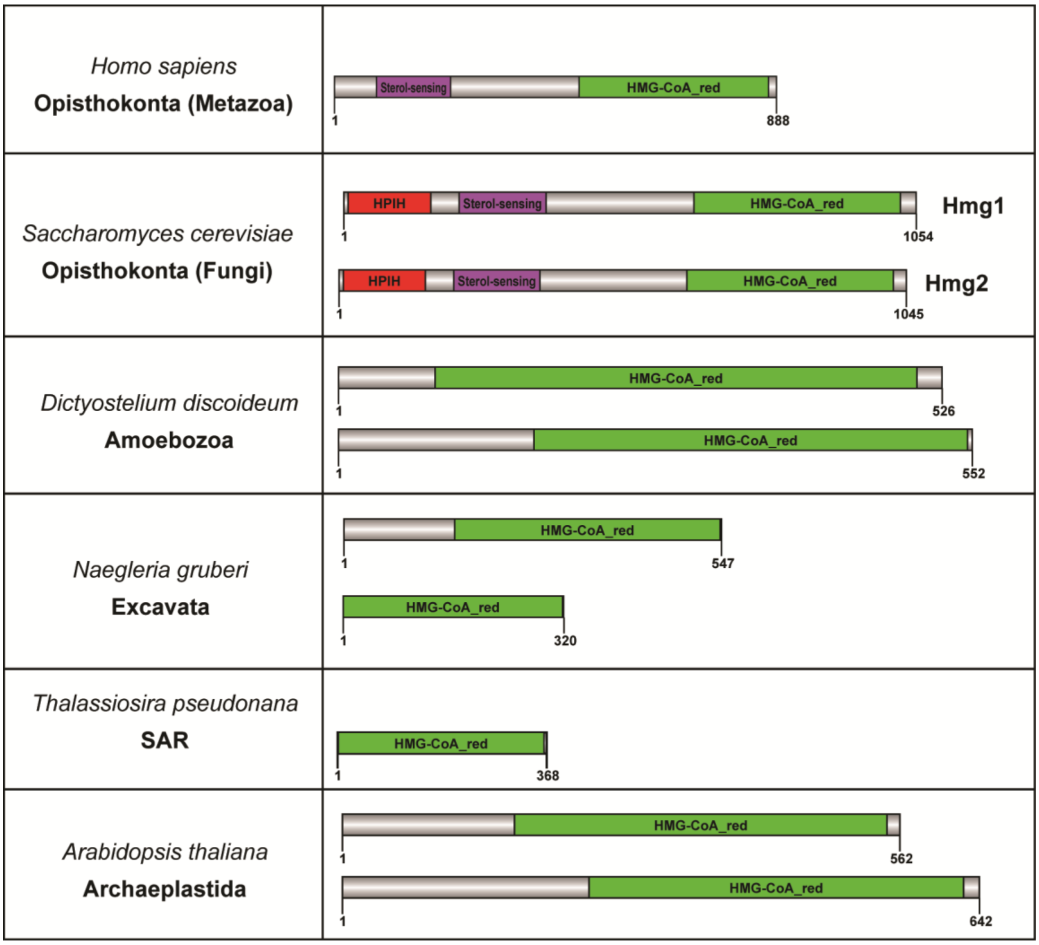

Figure S2: Domain organization in Hmg1 & Hmg2 proteins. Homologs of Hmg1 and Hmg2 proteins are shown in representative organisms. The domains found in each of the homologs are shown in different colors. HMG-CoA_red (green), sterol-sensing (purple), HPIH (red). The homolog in *Thalassiosira pseudonana* with HMG-CoA_red domain is partial. All maps are drawn to scale.

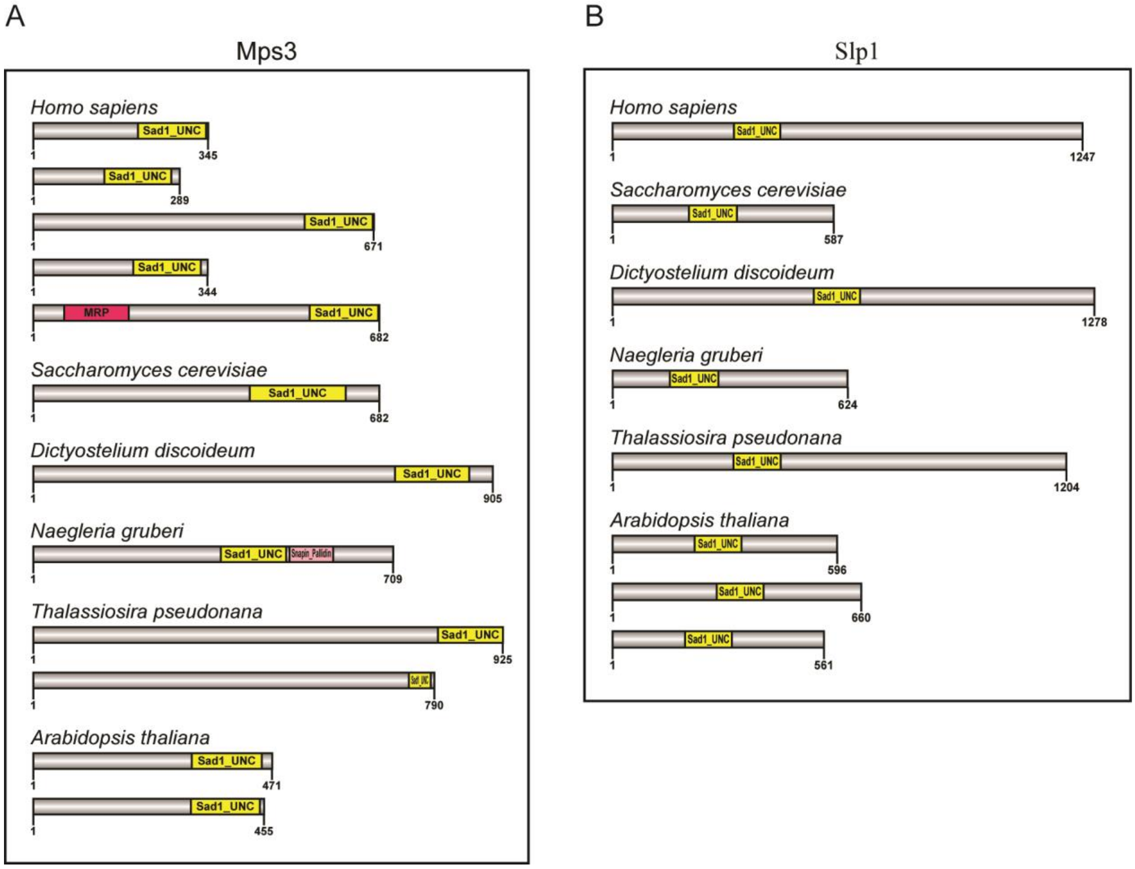

Figure S3: Domain organization in the SUN domain proteins. The domain architectures of the homologs of SUN domain containing proteins A) Mps3 (C-terminal SUN) and B) Slp1 (mid-SUN) are shown in representative organisms. Sad1_UNC domain is shown in yellow. The additional MRP domain found in Mps3 homolog of human is shown in dark pink.

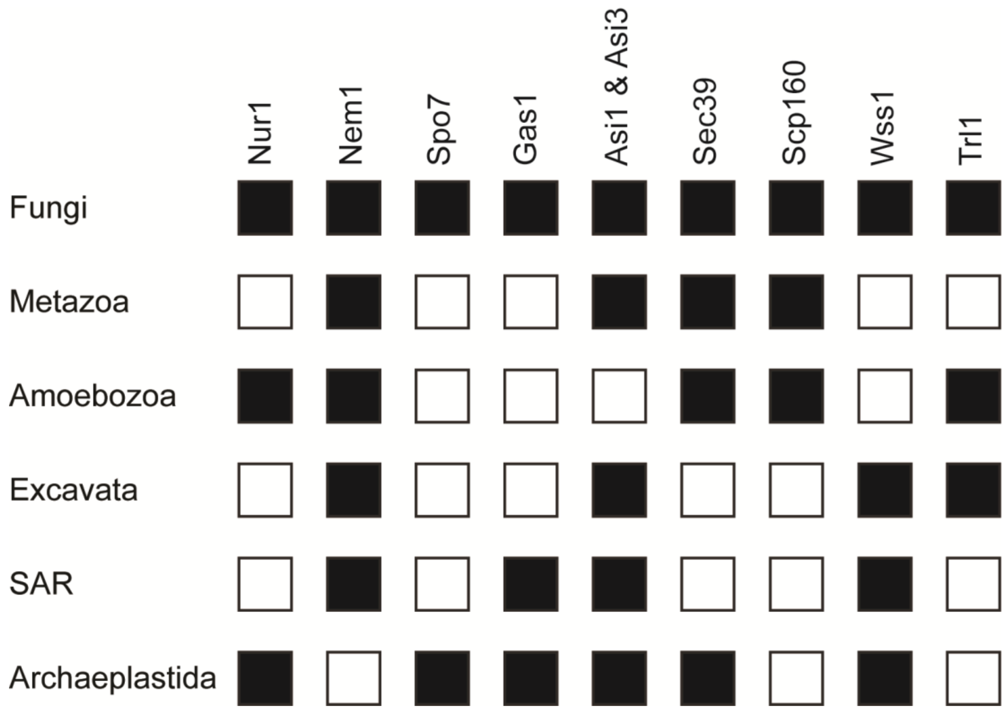

Figure S4: Non-linearly conserved proteins. The occurrence of non-linearly conserved proteins across supergroups is depicted. The black filled rectangles represent the presence of the protein in the respective supergroup. Fungi and Metazoa, belonging to Opisthokonta, are shown separately.

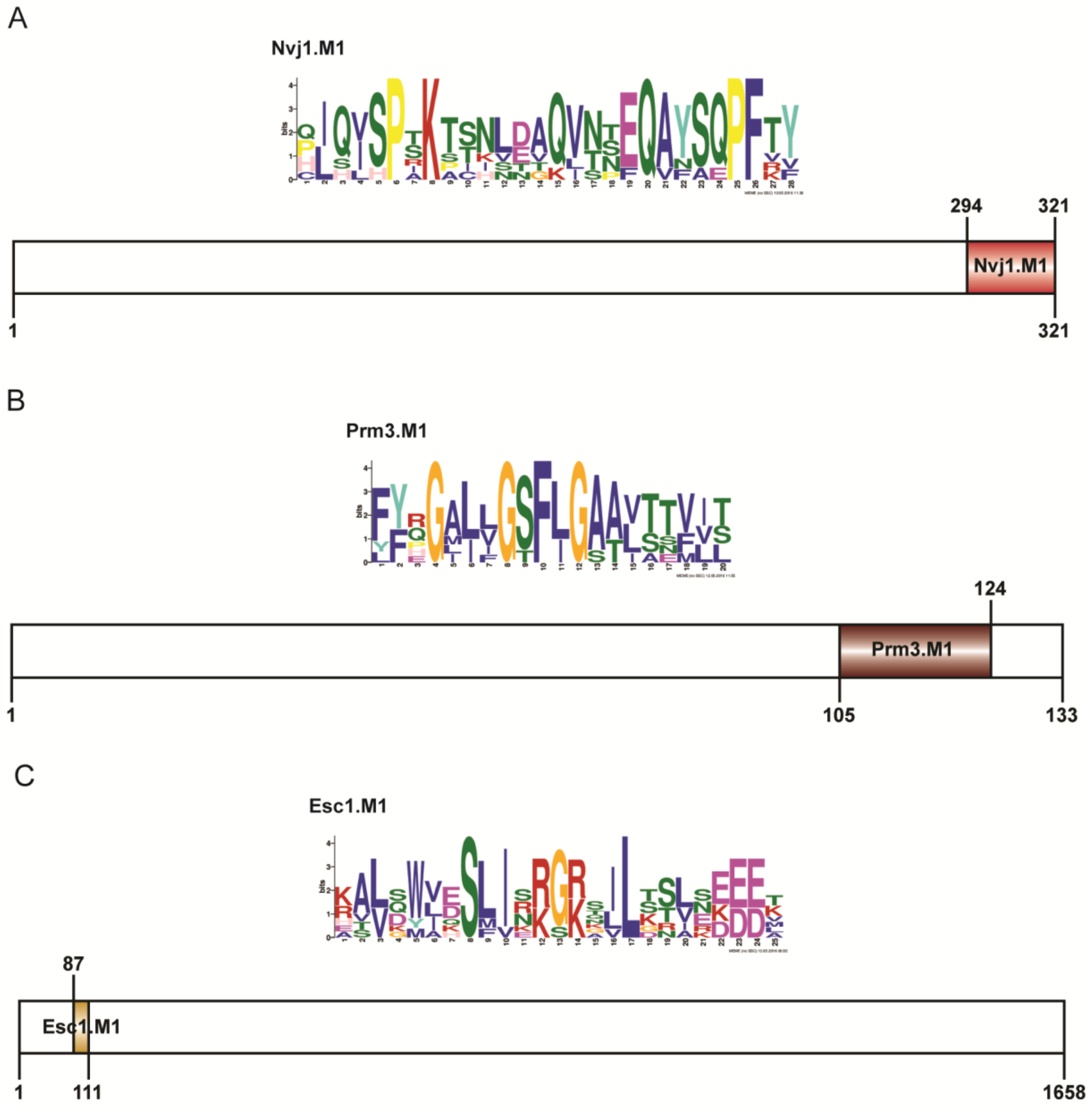

Figure S5: Motifs identified in Saccharomycetes specific proteins. The sequence logos of the motifs identified in A) Nvj1, B) Prm3 and C) Esc1 are shown. Shown below is the scaled map of the *S. cerevisiae* protein indicating the position of the motif.

Table S1: Nuclear envelope proteome of *Saccharomyces cerevisiae*

S. No	Protein	Functional class	Localisation
1	Ebp2	Chromatin organisation	Nuclear periphery
2	Rrs1	Chromatin organisation	Nuclear periphery
3	Mps3	Chromatin organisation	INM
4	Heh2	Chromatin organisation	INM
5	Src1	Chromatin organisation	INM
6	Nur1	Chromatin organisation	Nuclear periphery
7	Esc1	Chromatin organisation	Nuclear periphery
8	Hmg1	Nuclear envelope homeostasis	ER & ONM
9	Hmg2	Nuclear envelope homeostasis	ER & ONM
10	Pct1	Nuclear envelope homeostasis	ER & ONM
11	Nem1	Nuclear envelope homeostasis	ER & NE
12	Spo7	Nuclear envelope homeostasis	ER & NE
13	Brr6	Nuclear envelope homeostasis	ER & NE
14	Brl1	Nuclear envelope homeostasis	ER & NE
15	Apq12	Nuclear envelope homeostasis	ER & NE
16	Ulp1	Gene Regulation	Nuclear pore
17	Ssm4	Gene Regulation	ER & INM
18	Rrt12	Gene Regulation	ER & NE
19	Gas1	Gene Regulation	Nuclear periphery
20	Asi1	Gene Regulation	INM
21	Asi2	Gene Regulation	INM
22	Asi3	Gene Regulation	INM
23	Cse1	Transport related	Nuclear pore
24	Ntf2	Transport related	Nuclear pore
25	Thp1	Transport related	Nuclear pore
26	Pml39	Transport related	Nuclear pore
27	Sec39	Transport related	NE & ER
28	Pga2	Transport related	NE & ER
29	Has1	Others	NE
30	Ptc7	Others	INM
31	Trm1	Others	INM
32	Jem1	Others	ER & ONM
33	Slp1	Others	ER & ONM
34	Scp160	Others	ER & ONM
35	Wss1	Others	NE
36	Trl1	Others	INM
37	Gtt3	Others	Nuclear periphery
38	Uip4	Others	ER & NE
39	Mps2	Others	NE
40	Nbp1	Others	INM
41	Ypr174c	Others	NE
42	Nvj1	Others	NE

43	Prm3	Others	ONM
44	Cos8	Others	NE
45	Uip3	Others	NE

The proteins present at the nuclear envelope of *Saccharomyces cerevisiae* are listed along with the functional class to which they belong and their specific localization.

Table S2: List of organisms used in the study

S. No	Organism	Phylum/Class/Common name	Eukaryotic Supergroup	Proteome Downloaded on
1	*Caenorhabditis elegans*	Nematode	Opisthokonta	29/09/2015
2	*Drosophila melanogaster*	Fruit fly	Opisthokonta	29/09/2015
3	*Anopheles gambiae str. PEST*	Mosquito	Opisthokonta	29/09/2015
4	*Ciona intestinalis*	Tunicate	Opisthokonta	29/09/2015
5	*Danio rerio*	Zebrafish	Opisthokonta	29/09/2015
6	*Takifugu rubripes*	Pufferfish	Opisthokonta	29/09/2015
7	*Anolis carolinensis*	Green anole lizard	Opisthokonta	29/09/2015
8	*Gallus gallus*	Chicken	Opisthokonta	30/09/2015
9	*Ornithorhynchus anatinus*	Platypus	Opisthokonta	29/09/2015
10	*Monodelphis domestica*	Opossum	Opisthokonta	29/09/2015
11	*Canis lupus familiaris*	Dog	Opisthokonta	30/09/2015
12	*Sus scrofa*	Pig	Opisthokonta	30/09/2015
13	*Mus musculus*	Mouse	Opisthokonta	29/09/2015
14	*Pan troglodytes*	Chimpanzee	Opisthokonta	30/09/2015
15	*Homo sapiens*	Human	Opisthokonta	28/09/2015
16	*Strongylocentrotus purpuratus*	Sea urchin	Opisthokonta	29/09/2015
17	*Amphimedon queenslandica*	Sponge	Opisthokonta	29/09/2015
18	*Arthroderma otae CBS 113480*	Eurotiomycetes	Opisthokonta	28/09/2015
19	*Aspergillus nidulans FGSC A4*	Eurotiomycetes	Opisthokonta	28/09/2015
20	*Neosartorya fischeri NRRL 181*	Eurotiomycetes	Opisthokonta	28/09/2015
21	*Leptosphaeria maculans JN3*	Dothideomycetes	Opisthokonta	28/09/2015
22	*Parastagonospora nodorum SN15*	Dothideomycetes	Opisthokonta	28/09/2015
23	*Botrytis cinerea B05.10*	Leotiomycetes	Opisthokonta	28/09/2015

24	*Sclerotinia sclerotiorum 1980 UF-70*	Leotiomycetes	Opisthokonta	28/09/2015
25	*Chaetomium globosum CBS 148.51*	Sordariomycetes	Opisthokonta	28/09/2015
26	*Thielavia terrestris NRRL 8126*	Sordariomycetes	Opisthokonta	28/09/2015
27	*Neurospora crassa OR74A*	Sordariomycetes	Opisthokonta	28/09/2015
28	*Tuber melanosporum Mel28*	Pezizomycetes	Opisthokonta	28/09/2015
29	*Candida glabrata CBS 138*	Saccharomycetes	Opisthokonta	28/09/2015
30	*Zygosaccharomyces rouxii CBS 732*	Saccharomycetes	Opisthokonta	28/09/2015
31	*Kluyveromyces lactis NRRL Y-1140*	Saccharomycetes	Opisthokonta	28/09/2015
32	*Schizosaccharomyces pombe 972h-*	Schizosaccharomycetes	Opisthokonta	28/09/2015
33	*Agaricus bisporus var. bisporus H97*	Agaricomycetes	Opisthokonta	28/09/2015
34	*Schizophyllum commune H4-8*	Agaricomycetes	Opisthokonta	29/09/2015
35	*Trametes versicolor FP-101664 SS1*	Agaricomycetes	Opisthokonta	29/09/2015
36	*Auricularia subglabra TFB-10046 SS5*	Agaricomycetes	Opisthokonta	29/09/2015
37	*Cryptococcus neoformans var. grubii H99*	Tremellomycetes	Opisthokonta	29/09/2015
38	*Ustilago maydis 521*	Ustilaginomycetes	Opisthokonta	29/09/2015
39	*Puccinia graminis f. sp. tritici CRL 75-36-700-3*	Pucciniomycetes	Opisthokonta	29/09/2015
40	*Batrachochytrium dendrobatidis JAM81*	Chytridiomycota	Opisthokonta	29/09/2015
41	*Encephalitozoon intestinalis ATCC 50506*	Microsporidia	Opisthokonta	29/09/2015
42	*Entamoeba histolytica HM-1:IMSS*	Archamoebae	Amoebozoa	16/09/2015
43	*Acytostelium subglobosum LB1*	Mycetozoa	Amoebozoa	28/09/2017
44	*Dictyostelium discoideum AX4*	Mycetozoa	Amoebozoa	14/09/2015
45	*Polysphondylium pallidum PN500*	Mycetozoa	Amoebozoa	28/09/2017
46	*Trypanosoma brucei gambiense DAL972*	Kinetoplastid	Excavata	28/09/2015
47	*Leishmania major strain Friedlin*	Kinetoplastid	Excavata	28/09/2015
48	*Naegleria gruberi strain NEG-M*	Heterolobosean	Excavata	28/09/2015
49	*Trichomonas vaginalis G3*	Parabasalid	Excavata	28/09/2015
50	*Giardia lamblia ATCC 50803*	Diplomonad	Excavata	28/09/2015
51	*Plasmodium falciparum 3D7*	Apicomplexan	SAR	13/04/2017
52	*Theileria parva strain Muguga*	Apicomplexan	SAR	28/09/2015
53	*Babesia bovis T2Bo*	Apicomplexan	SAR	28/09/2017
54	*Toxoplasma gondii ME49*	Apicomplexan	SAR	13/04/2017
55	*Cryptosporidium hominis TU502*	Apicomplexan	SAR	28/09/2015

56	*Paramecium tetraurelia strain d4-2*	Ciliate	SAR	28/09/2017
57	*Tetrahymena thermophila SB210*	Ciliate	SAR	16/09/2015
58	*Phytophthora infestans T30-4*	Oomycete	SAR	16/09/2015
59	*Phaeodactylum tricornutum CCAP 1055/1*	Diatom	SAR	16/09/2015
60	*Thalassiosira pseudonana CCMP1335*	Diatom	SAR	16/09/2015
61	*Bigelowiella natans CCMP2755*	Rhizaria	SAR	02/11/2017
62	*Cyanophora paradoxa*	Glaucophyta	Archaeplastida	27/10/2017
63	*Chondrus crispus*	Red alga	Archaeplastida	03/10/2017
64	*Cyanidioschyzon merolae strain 10D*	Red alga	Archaeplastida	16/10/2017
65	*Chlamydomonas reinhardtii*	Green alga	Archaeplastida	30/09/2015
66	*Volvox carteri f. nagariensis*	Green alga	Archaeplastida	16/10/2017
67	*Physcomitrella patens*	Moss	Archaeplastida	16/10/2017
68	*Marchantia polymorpha subsp. ruderalis*	Liverwort	Archaeplastida	18/10/2017
69	*Amborella trichopoda*	Basal Angiosperm	Archaeplastida	18/10/2017
70	*Oryza sativa Japonica Group*	Rice	Archaeplastida	30/09/2015
71	*Zea mays*	Maize	Archaeplastida	16/10/2017
72	*Arabidopsis thaliana*	Thale cress	Archaeplastida	30/09/2015
73	*Glycine max*	Soybean	Archaeplastida	16/10/2017

The table includes the list of organisms chosen for this study along with the phylum/class/common name, the eukaryotic supergroup to which the organism belongs and the date on which the proteomes were downloaded.
